# Supplementary material for: Angiogenic CD8 T cells from PWH induce Granzymes-dependent PAR1 activation promoting endothelial inflammation
Source: bioRxiv. 2025 Sep 22:2025.08.06.668764. Preprint. [Version 2] doi: 10.1101/2025.08.06.668764 (PMC12478364; doi:10.1101/2025.08.06.668764)
Supplement: Supplement 1 [file media-1.pdf]

Supplementary Materials

Angiogenic CD8 T cells from PWH induce Granzymes-dependent PAR1 activation promoting endothelial inflammation

Tong Li[1], Chinmayee Mehta[1], Cooper James[1], Jinya Chen[1], Hui Chen[1], Gerard P Ahern[2], Ziang Zhu[1], Maitane Faus Cid[1], Maryam Abdussamad[1], Shiwen Yu, Jai Kumar[3], Princy Kumar[3], Colleen Hadigan[4], Kyle Di Vito[5], Dorian B McGavern[6], Leonid Pobezinsky[7], and Marta Catalfamo[1]

Figure S1. Phenotypic characterization of lymphoid organs from naïve C57BL/6-F2r$^{fl/fl}$ mice. **(A)** Scheme of the Exon 2 of F2r gene conditionally targeted. **(B)** Live cells and singlets were pre-gated followed by analysis of total hematopoietic cells (CD45^{+}) to assess cell counts in lymph nodes (LNs) and spleen from C57BL/6-F2r$^{fl/fl}$ (n= 5, open red symbol) and C57BL/6 (n= 5, open black symbol). **(C)** Gating strategy of immune cell subsets (CD45^{+}): NK cells (CD3^{-}NK1.1^{+}), T cells (CD3^{+}), Eosinophils (Eos, SSC-A^{high}Gr1^{int}), Neutrophils (Neut, Gr1^{+}), MHC-II^{+} monocyte/macrophage (R1: B220^{-}MHC-II^{+}CD11b^{+}), B cells (R2: MHCII^{+}B220^{+}), plasmacytoid dendritic cells (pDCs) (R3: B220^{+}MHCII^{-}CD11c^{+}). MHCII^{-} monocyte/macrophage (R4: B220^{-}MHCII^{-}CD11b^{+}), conventional dendritic cells (cDCs) (R1: B220^{-}MHCII^{+}CD11c^{+}). **(D)** Percentage and cell count of myeloid subsets (Eos, Neut, cDCs, pDCs and monocyte/macrophage) in LNs and spleen. **(E)** Percentage and cell count of lymphocytes B cells, NK cells and T cells in LNs and spleen. The bar graph is represented by box and whisker showing the median value with first and third quartiles in the box, with whiskers extending to the minimum and maximum values. Statistical analysis was performed using non-parametric Mann-Whitney test. *P* value <0.05 was considered significant.

Figure S2. Expression of PAR1 in CD4 and CD8 T cells.

(A) Splenic cells from C57BL/6-F2r$^{fl/fl}$ mice were isolated and activated *in vitro* with anti-CD3/CD28 mAbs for 3 days and expanded with IL-2 for an additional 5 days. PAR1 expression was assessed in resting and activated T cells by flow cytometry using rabbit anti-PAR1 mAb (Abcam, catal. ab322457) followed by an Alexa 647 labeled polyclonal anti-rabbit IgG. Contour plots of tdTomato and PAR1 expression by resting and *in vitro* activated CD4 and CD8 T cells.

(Lower panel**)** Histogram of PAR1 expression by resting and *in vitro* activated CD4 and CD8 T cells.

Figure S3. PAR1 expression in lung ECs and Granzyme A expression by CD8 T cells in acute LCMV infection

(A) C57BL/6-F2r$^{fl/fl}$ mice were infected with LCMV Armstrong (Arm) strain (2 x 10^5 pfu, i.v). Day 6 and 21 p.i., splenic and lung immune cells were isolated and stimulated *in vitro* with LCMVGp33-41 peptide for 4 hours. DMSO was used as control. Representative contour plots of IFNγ and TNFα expression by CD8 T cells. Frequency of splenic and lung CD8 T cells secreting IFNγ and TNFα from naïve (N) mice and infected mice day 6 and day 21 p.i. **(B)** Representative contour plots of IFNγ and CD107a expression by CD8 T cells. Frequency of total (blue gate) CD107a$^+$ CD8 T cells in infected mice day 6 and day 21 p.i Naïve (N) animals were used as controls. **(C)** Median Fluorescence Intensity (MFI) of tdTomato expression in splenic and lung total CD8 T cells from naïve (N) and infected mice at day 6 and 21 p.i. **(D)** Representative contour plots of GMZA in degranulating (CD107a$^+$) LCMV-specific CD8 T cells from spleen and lung. at day 6 p.i. Naïve (N) animals were used as controls. **(E)** Median fluorescence intensity of PAR1 (tdT$^+$) expression in CD107a$^+$ and CD107$^-$ LCMV-specific CD8 T cells. The data in line graphs represent mean±SEM in each time point. Data are pooled from three independent experiments with 3-5 naïve animals and 5-17 per time point (F and H). The bar graph is represented by box and whisker showing the median value with first and third quartiles in the box, with whiskers extending to the minimum and maximum values. Statistical analysis was performed using non-parametric Mann-Whitney test. *P* value < 0.05 was considered significant.

Figure S4. Expression of PAR1 in CD31^{high}CD8 T cells during LCMV infection.

(A) C57BL/6-F2r$^{fl/fl}$ mice were infected with LCMV Armstrong (Arm) strain (2 x 10^5 pfu, i.v). At day 15 p.i., mice were administered anti-CD8α mAb and IgG2b isotype for control mice. LCMV *glycoprotein* (*Gp*) mRNA expression was assessed in spleen and lung at day 6 and 21 p.i, and in mice treated with IgG and anti-CD8 mAb at day 21 p.i. Spleen collected from mice at day 3 p.i. was used as positive control (red dash line). **(B)** Representative contour plot of CD31^{high} and CD309 expression by lung CD8 T cells from naïve mice and infected mice. Frequency and cell count of CD31^{high}CD8 T cells from naïve (N) mice and infected mice at day 6 and 21 p.i. Median fluorescence intensity of PAR1 (tdT^{+}) expression in CD31^{high} and CD31^{high} CD8 T cells. **(C)** Lung CD31^{high}CD8 and CD31^{neg}CD8 T cells LCMVArm infected C57BL/6-F2r$^{fl/fl}$ mice were sorted at day 21 p.i. CD31^{high}CD8 T cells were sorted from Naïve (N) mice (n= 3). Sorted T cells were stimulated *in vitro* in anti-CD3 and anti-CD28 mAbs coated plate. After 3 days stimulation cytokines were measured in the supernatants. Detection of growth factors macrophage colony-stimulating factor (M-CSF), platelet derived growth factor-A (PDGF-A) and granulocyte macrophage colony stimulating factor (GM-CSF), IL-2, IL-10, Th2 cytokines (IL-4, IL-5 and IL-13), Th17 cytokines (IL-17A and IL-6), Th22 cytokines (IL-22), Th9 (IL-9). The graph is represented by box and whisker showing the median value with first and third quartiles in the box, with whiskers extending to the minimum and maximum values. The data in line graphs represent mean±SEM in each time point. Data are pooled from two independent experiments with 3 naïve animals and 5-10 per time point. Statistical analysis was performed using non-parametric Mann-Whitney test. *P* value < 0.05 was considered significant.

Figure S5. Unsupervised clustering analysis of lung endothelial cells during LCMV infection.

C57BL/6-F2r$^{fl/fl}$ mice were infected with LCMVArm strain and lung cells were isolated from naïve and infected mice at day 6 and 21 p.i and analyzed by flow cytometry expression of CD31, PDL1, tdTomato, CD34, CD309, VCAM-1, CD105, CD90.2 and CD201. 10,000 of manually gated lung endothelial cells (CD326$^-$CD45$^-$CD31$^+$) were concatenated from each naïve mice and infected mice at day 6 and 21 p.i. Unsupervised clusters were analyzed with Phenograph (k= 100). Heatmap of mean fluorescence intensity of each marker for all the clusters and bubble plots of median frequency of each cluster from naïve and infected mice (Figure 3). Frequency of each cluster is represented by box and whisker showing the median value with first and third quartiles in the box, with whiskers extending to the minimum and maximum values. Statistical significance was performed using non-parametric Mann-Whitney test. *P* value <0.05 was considered significant.

Figure S6. Relationship between plasma levels of GZMA and T cell derived cytokines.

(A) Relationship between plasma levels of CD8 T cell cytokines (IFNγ, TNFα, sFasL and IL-2) and granzymes (granzyme A and B) in PWH (n= 25). **(B)** *In vitro* activation and cytokine secretion by HUVEC. 5,000 HUVEC were stimulated with 20 ng/mL of rhIFNγ and 0.5 ng/mL of rhTNFα overnight and cytokines were measured in the supernatants. Detection of soluble adhesion molecules sVCAM-1 and sICAM-1 and cytokines IL-6, VEGF, Angiopoietin-2, FGFb, and IL-8. **(C)** *In vitro* HUVEC activation with granzymes. HUVEC were stimulated overnight with 100 nM of rhGZMA and rhGZMK in s the positive control, HUVEC were incubated with 100 μM of PAR1 agonist peptide (TFLLR-NH2) and control peptide (RLLFT-NH2). IL-6, IL-8 and Ang-2 were measured in the supernatants. Data is represented by box and whisker showing the median value with first and third quartiles in the box, with whiskers extending to the minimum and maximum values. Statistical significance was performed using non-parametric Mann-Whitney test. *P* value

<0.05 was considered significant. Correlations were performed using nonparametric Spearman correlation and *p* value $\leqslant$ 0.01 was considered significant.

Figure S7. PAR1 dependent activation by GZMA and GZMK. HUVEC were stimulated overnight with 100 nM of rhGZMA and rhGZMK in the presence or absence of 10 μg/mL of ATAP-2 mAb or 300 nM of PAR1 antagonist SCH530348. As the positive control, HUVEC were incubated with 100 μM of PAR1 agonist peptide (TFLLR-NH2) and control peptide (RLLFT-NH2). VEGF, bFGF, sVCAM-1, sICAM-1 and sPCAM were measured in the supernatants. Data is represented by box and whisker showing the median value with first and third quartiles in the box, with whiskers extending to the minimum and maximum values. Statistical significance was performed using non-parametric Mann-Whitney test. *P* value <0.05 was considered significant.

Table S1. Mouse Flow cytometry panels

Flow cytometry panel-1 (Endothelial cells staining)

Specificity	Fluorochrome	Clone	Cat. #	Manufacturer
CD90.2	BV570	30-H12	105329	BioLegend
CD34	PerCP-Cy5.5	MEC14.7	119327	BioLegend
CD105	Pacific Blue	MJ7/18	120412	BioLegend
CD326	BV510	G8.8	118231	BioLegend
CD31	BV605	390	102427	BioLegend
CD45	BV711	53-6.7	103147	BioLegend
PD-L1	BV785	10F.9G2	124331	BioLegend
VCAM-1	FITC	429 (MVCAM.A)	105706	BioLegend
CD309	PE-Vio770	REA1116	130-119-436	Miltenyi Biotech
CD16/CD32	N/A	2.4G2	553141	BD Bioscience

Flow cytometry panel-2 (Phenotypic characterization of lymphoid organs)

Specificity	Fluorochrome	Clone	Cat. #	Manufacturer
B220	BV510	RA3-6B2	103247	Biolegend
CD4	BV605	RM4-5	100547	Biolegend
MHC II	BV655	M5/114.15.2	107641	Biolegend
CD45	BV711	30-F11	103147	Biolegend
CD11c	BV750	N418	117357	Biolegend
Gr-1	FITC	RB6-8C5	108406	Biolegend
CD3	BUV805	17A2	569192	BD
CD11b	BUV737	M1/70	564443	BD
CD8a	BUV496	53-6.7	569181	BD
NK 1.1	AF700	S17016D	156512	Biolegend
CD16/CD32	N/A	2.4G2	553141	BD Bioscience

Flow cytometry panel-3 (LCMV-specific T cell staining)

Specificity	Fluorochrome	Clone	Cat. #	Manufacturer
CD107a	BV785	1D4B	121641	BioLegend
CD45	V570	30-F11	103136	BioLegend
CD31	V605	390	102427	BioLegend
CD127	BV650	A7R34	135043	BioLegend
CD44	BV750	IM7	103079	BioLegend
CD3	BUV805	17A2	569192	BD Bioscience
CD8a	BUV496	53-6.7	569181	BD Bioscience
KLRG1	PE-Cy7	2F1/KLRG1	138416	BioLegend
CD16/CD32	N/A	2.4G2	553141	BD Bioscience
IFNγ	APC	XMG1.2	562018	BD Bioscience
TNFα	PerCP/Cy5.5	MP6-XT22	506322	Biolegend
GZMA	eFluor450	3G8.5	48-5831-82	eBioscience

Table S2. Flow cytometry panels

Flow cytometry panel-1 (PBMC staining)

Specificity	Fluorochrome	Clone	Cat. #	Manufacturer
CD31	BUV395	133.1	745600	BD Bioseince
CD4	BUV805	SK3	564910	BD Bioseince
CD3	BV510	UCHT1	563109	BD Bioseince
CD8	BV605	SK1	564115	BD Bioseince
CD49f	BV650	GoH3	563706	BD Bioseince
PAR1	PE	WEDE15	IM2584	Beckmen Coulter
CXCR4	APC	12G5	560936	BD Bioseince
CX3CR1	APC-CY7	2A9-1	341615	Biolegend
GZMA	AF700	CB9	507210	Biolegend
GZMB	BV421	GB11	563389	BD Bioseince

Flow cytometry panel-2 (sorting HIV-specific CD8 T cells)

Specificity	Fluorochrome	Clone	Cat. #	Manufacturer
CD31	PE-Dazzle 594	WM59	303130	Biolegend
CD45RA	V605	HI100	562886	BD Bioseince
CD27	B515	M-T271	564642	BD Bioseince
CD3	V650	UCHT1	300468	Biolegend
CD8	PE-Cy7	SK1	344750	Biolegend
CXCR4	APC	12G5	560936	BD Bioseince
CD137	PE	4B4-1	561701	BD Bioseince

Figure S1

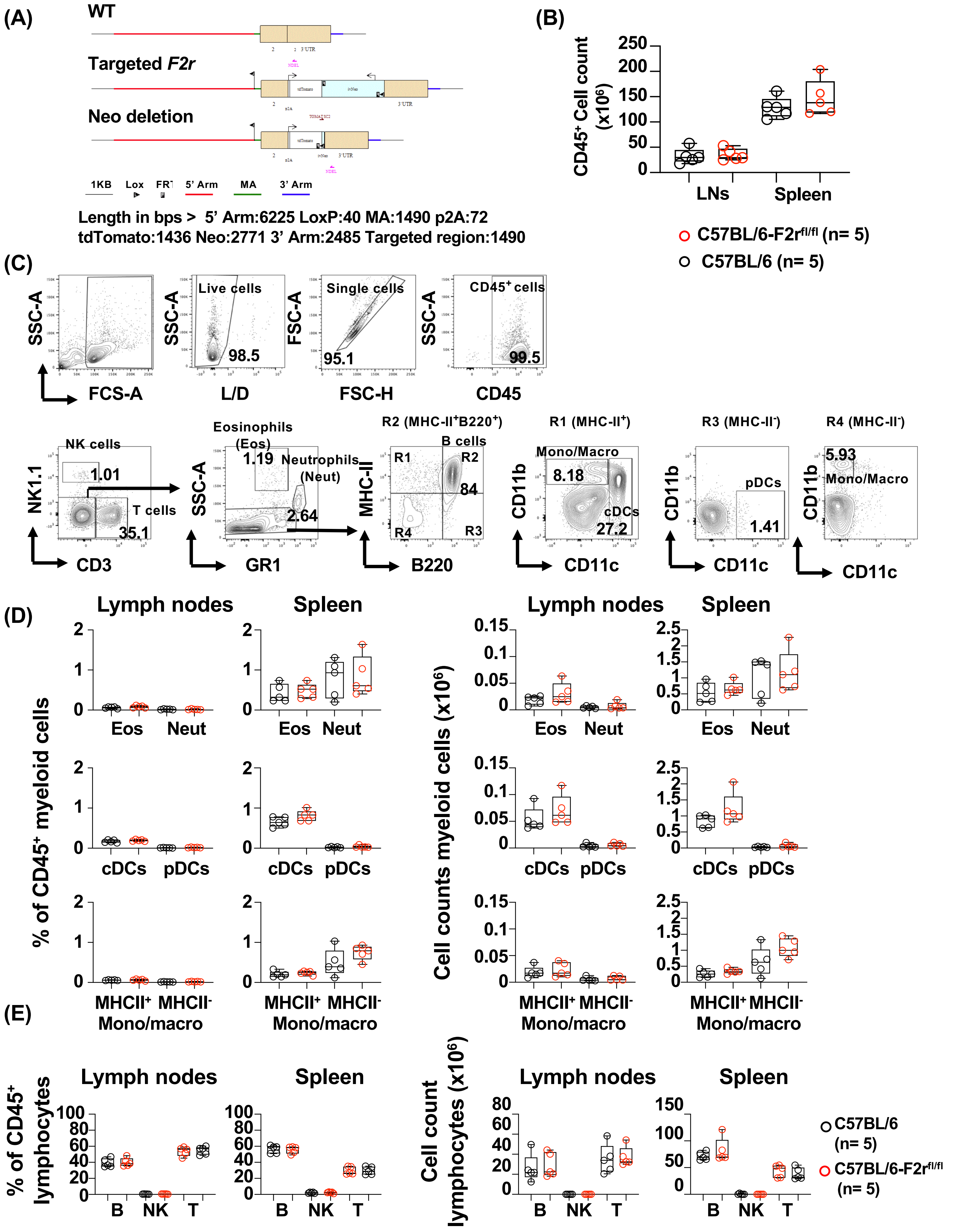

(A)
WT
Targeted F2r
Neo deletion
1KB
Lox
5' Arm
MA
3' Arm
Length in bps > 5' Arm:6225 LoxP:40 MA:1490 p2A:72
tdTomato:1436 Neo:2771 3' Arm:2485 Targeted region:1490
(B)
CD45+ Cell count (x10^6)
250
200
150
100
50
0
LNs
Spleen
C57BL/6-F2rfl/fl (n= 5)
C57BL/6 (n= 5)
(C)
SSC-A
FCS-A
Live cells
98.5
L/D
FSC-A
Single cells
95.1
FSC-H
CD45+ cells
99.5
CD45
NK cells
1.01
NK1.1
T cells
35.1
CD3
Eosinophils
(Eos)
1.19
Neutrophils
(Neut)
2.64
GR1
R2 (MHC-II+B220+)
B cells
R1
R2
84
R4
R3
MHC-II
B220
R1 (MHC-II+)
Mono/Macro
8.18
cDCs
27.2
CD11b
CD11c
R3 (MHC-II-)
pDCs
1.41
R4 (MHC-II-)
5.93
Mono/Macro
(D)
Lymph nodes
Spleen
% of CD45+ myeloid cells
Cell counts myeloid cells (x10^6)
Eos
Neut
cDCs
pDCs
MHCII+ MHCII-
Mono/macro
(E)
% of CD45+ lymphocytes
Cell count lymphocytes (x10^6)
B
NK
T
C57BL/6 (n= 5)
C57BL/6-F2rfl/fl (n= 5)

Figure S2

(A)

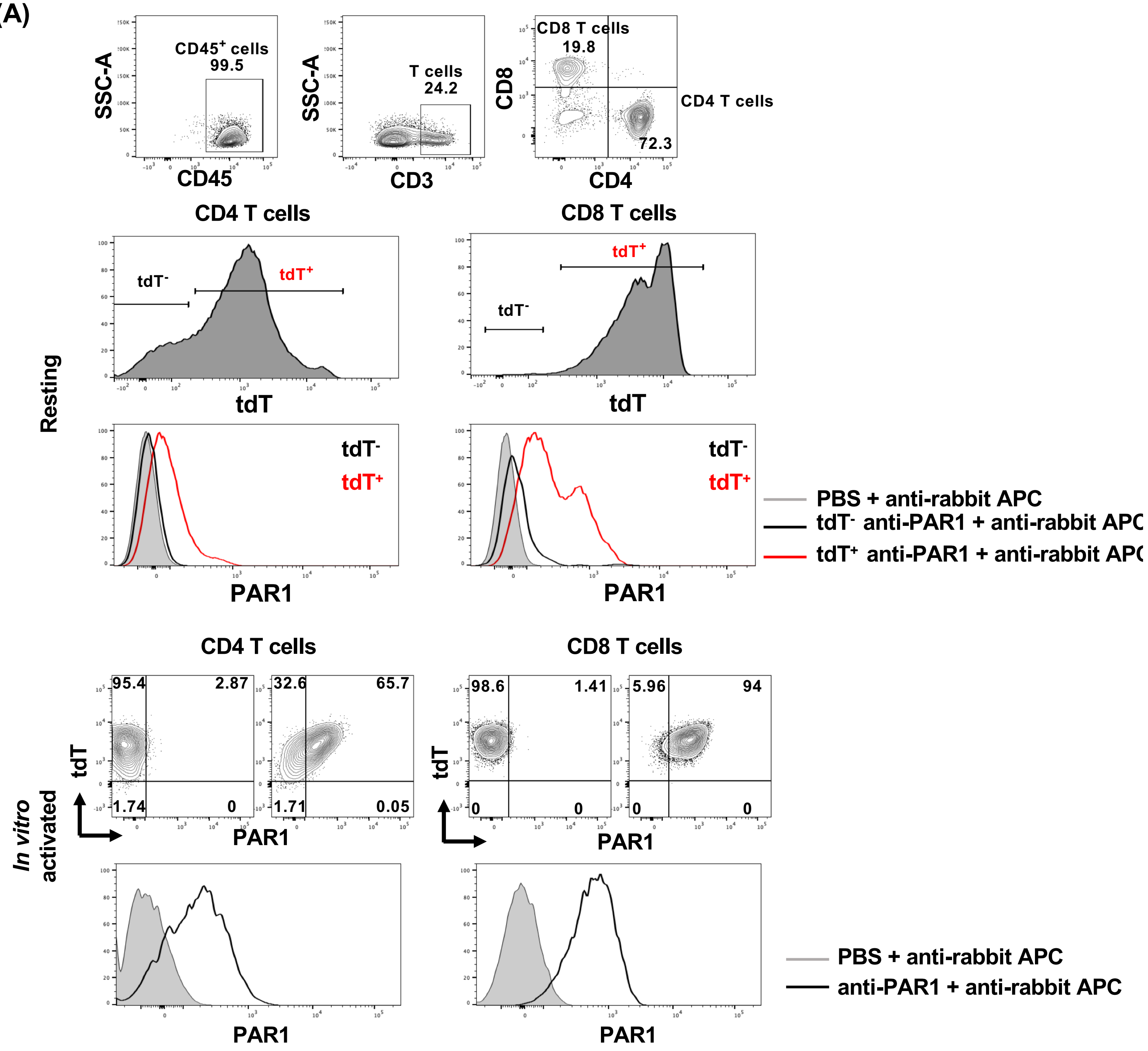

CD45+ cells
99.5
SSC-A
CD45
T cells
24.2
SSC-A
CD3
CD8 T cells
19.8
CD4 T cells
72.3
CD8
CD4
CD4 T cells
CD8 T cells
Resting
tdT-
tdT+
tdT
tdT-
tdT+
tdT
tdT-
tdT+
PAR1
tdT-
tdT+
PAR1
PBS + anti-rabbit APC
tdT- anti-PAR1 + anti-rabbit APC
tdT+ anti-PAR1 + anti-rabbit APC
CD4 T cells
CD8 T cells
95.4
2.87
1.74
0
32.6
65.7
1.71
0.05
98.6
1.41
0
0
5.96
94
0
0
tdT
PAR1
tdT
PAR1
In vitro activated
PAR1
PAR1
PBS + anti-rabbit APC
anti-PAR1 + anti-rabbit APC

Figure S3

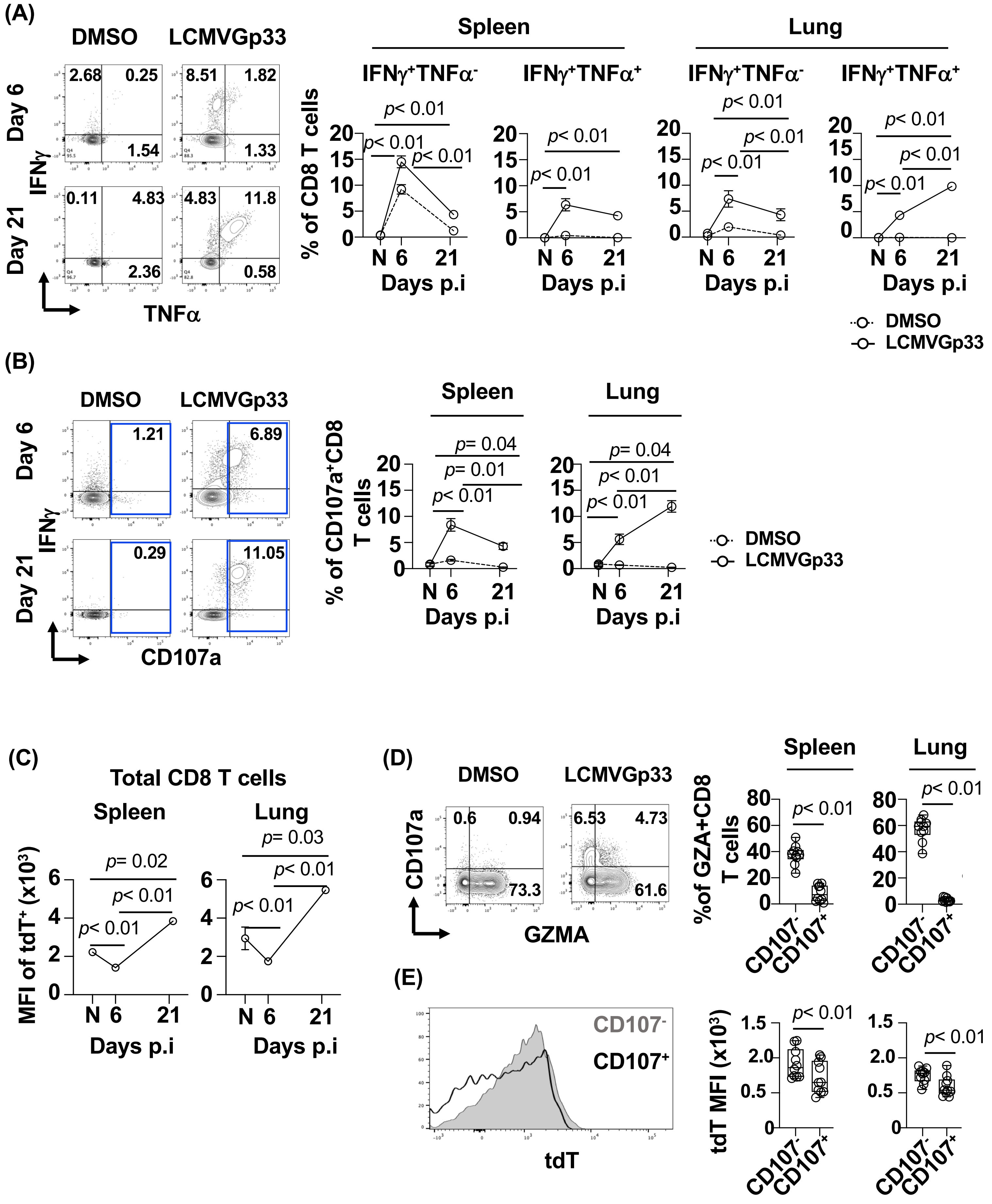
(A)
DMSO
LCMVGp33
Day 6
Day 21
IFNγ
TNFα
2.68
0.25
1.54
8.51
1.82
1.33
0.11
4.83
2.36
4.83
11.8
0.58
Spleen
Lung
IFNγ+TNFα-
IFNγ+TNFα+
% of CD8 T cells
Days p.i
DMSO
LCMVGp33
(B)
CD107a
1.21
6.89
0.29
11.05
% of CD107a+CD8 T cells
(C)
Total CD8 T cells
MFI of tdT+ (x10³)
(D)
CD107a
GZMA
0.6
0.94
73.3
6.53
4.73
61.6
%of GZA+CD8 T cells
CD107-
CD107+
(E)
tdT
tdT MFI (x10³)

Figure S4

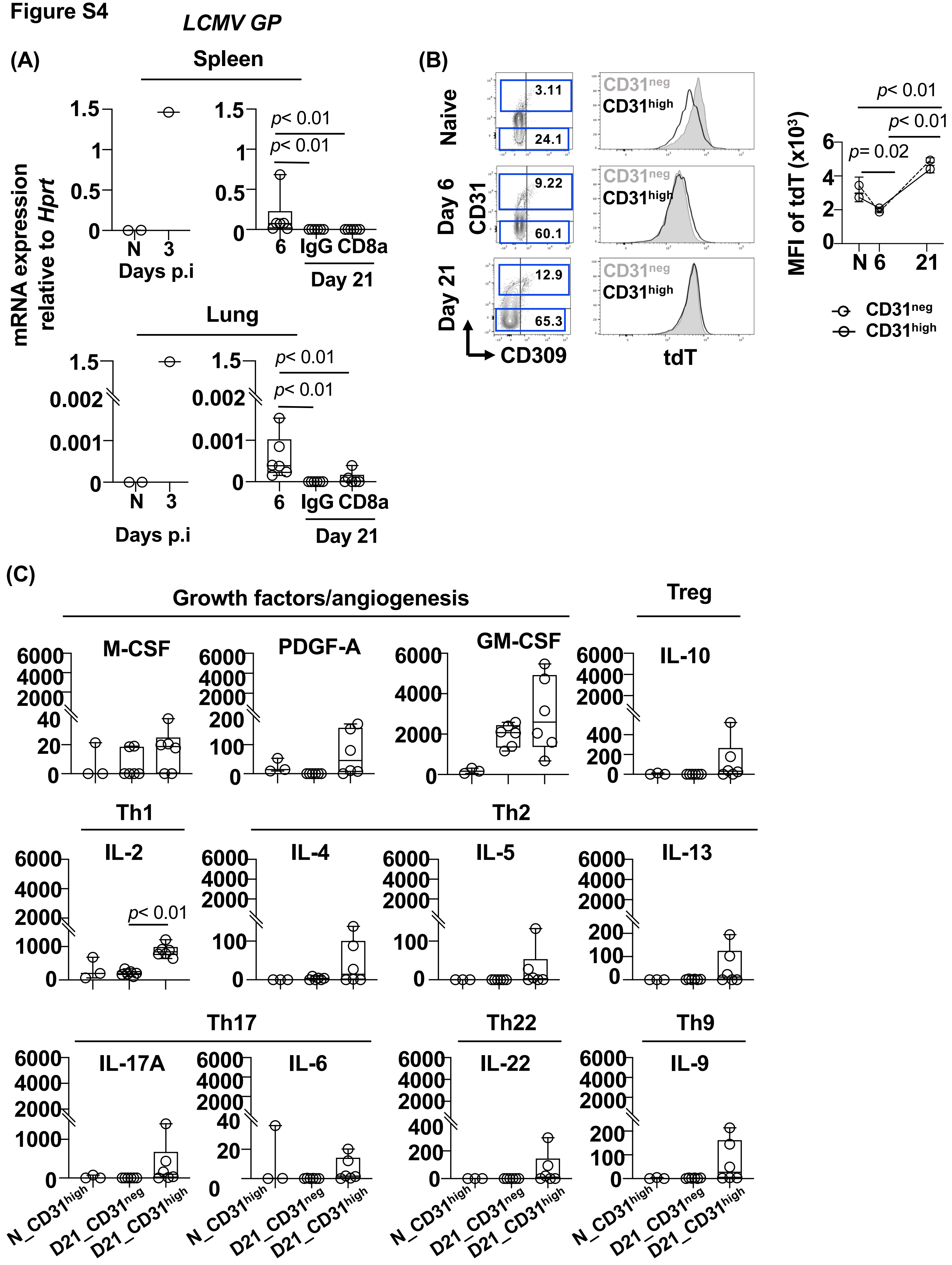
LCMV GP
(A)
Spleen
Lung
mRNA expression relative to Hprt
Days p.i
Day 21
N
3
6
IgG
CD8a
p< 0.01
(B)
Naive
Day 6
Day 21
CD31
CD309
tdT
CD31neg
CD31high
3.11
24.1
9.22
60.1
12.9
65.3
MFI of tdT (x10³)
p< 0.01
p= 0.02
(C)
Growth factors/angiogenesis
Treg
M-CSF
PDGF-A
GM-CSF
IL-10
Th1
Th2
IL-2
IL-4
IL-5
IL-13
Th17
Th22
Th9
IL-17A
IL-6
IL-22
IL-9
N_CD31high
D21_CD31neg
D21_CD31high

Figure S5

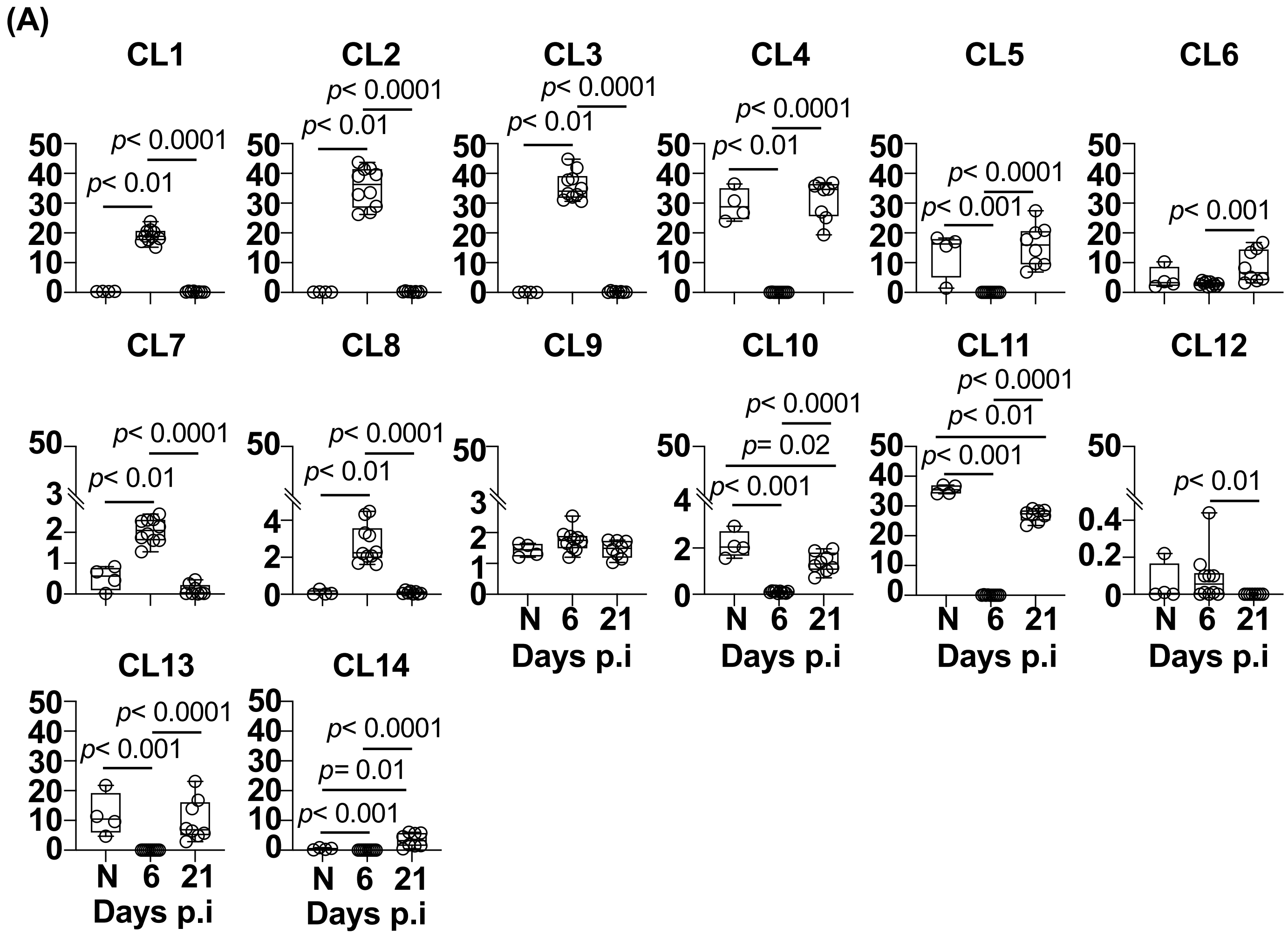
(A)
CL1
CL2
CL3
CL4
CL5
CL6
CL7
CL8
CL9
CL10
CL11
CL12
CL13
CL14
p< 0.01
p< 0.0001
p< 0.001
p= 0.02
p= 0.01
N
6
21
Days p.i

Figure S6

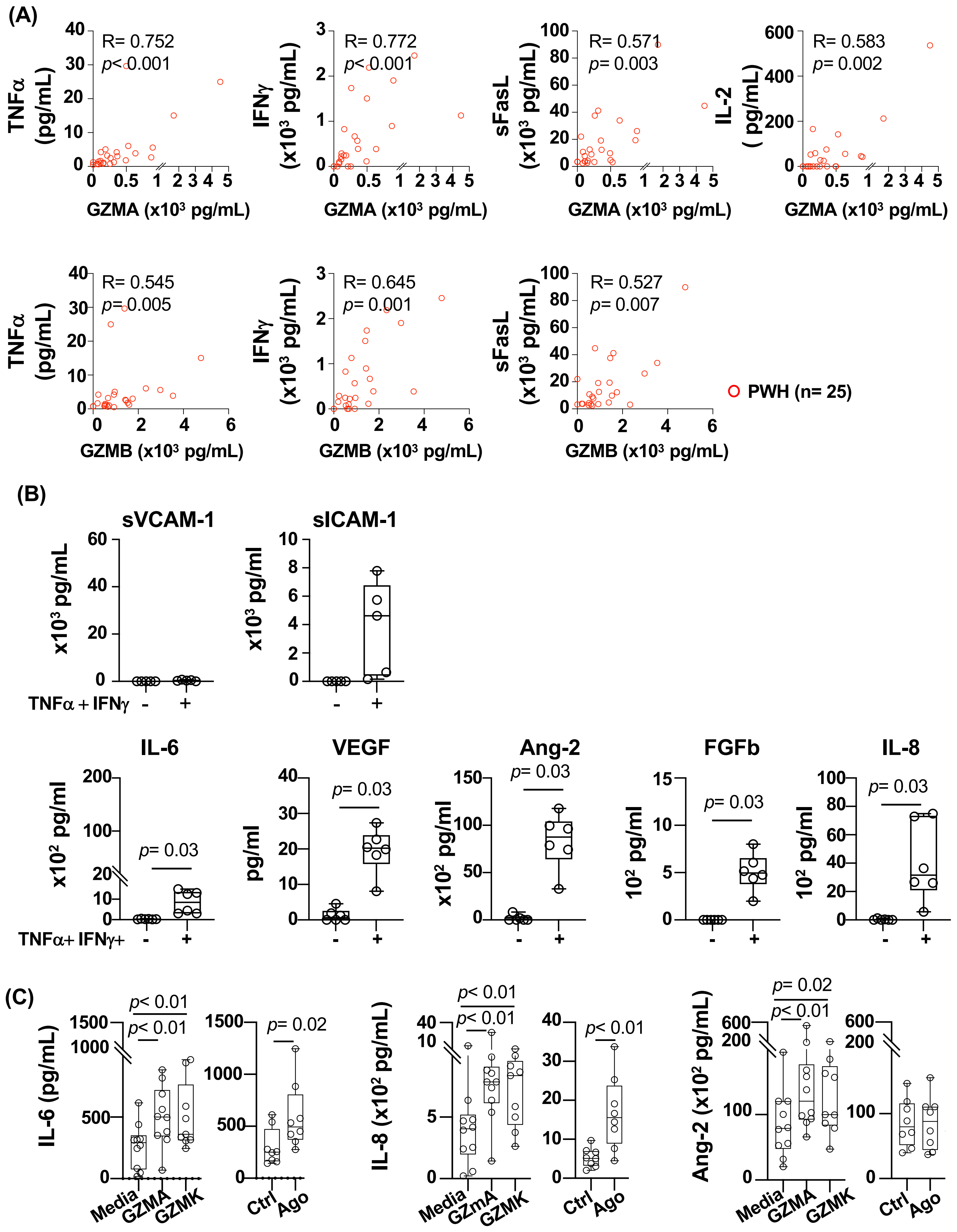
(A)
R= 0.752
p< 0.001
TNFα (pg/mL)
GZMA (x10³ pg/mL)
R= 0.772
p< 0.001
IFNγ (x10³ pg/mL)
R= 0.571
p= 0.003
sFasL (x10³ pg/mL)
R= 0.583
p= 0.002
IL-2 (pg/mL)
R= 0.545
p= 0.005
GZMB (x10³ pg/mL)
R= 0.645
p= 0.001
R= 0.527
p= 0.007
PWH (n= 25)
(B)
sVCAM-1
sICAM-1
x10³ pg/mL
x10³ pg/ml
TNFα + IFNγ
IL-6
VEGF
Ang-2
FGFb
IL-8
x10² pg/ml
pg/ml
10² pg/ml
p= 0.03
TNFα+ IFNγ+
(C)
IL-6 (pg/mL)
p< 0.01
p= 0.02
Media
GZMA
GZMK
Ctrl
Ago
IL-8 (x10² pg/mL)
GZmA
Ang-2 (x10² pg/mL)
p= 0.02

Figure S7

(A)

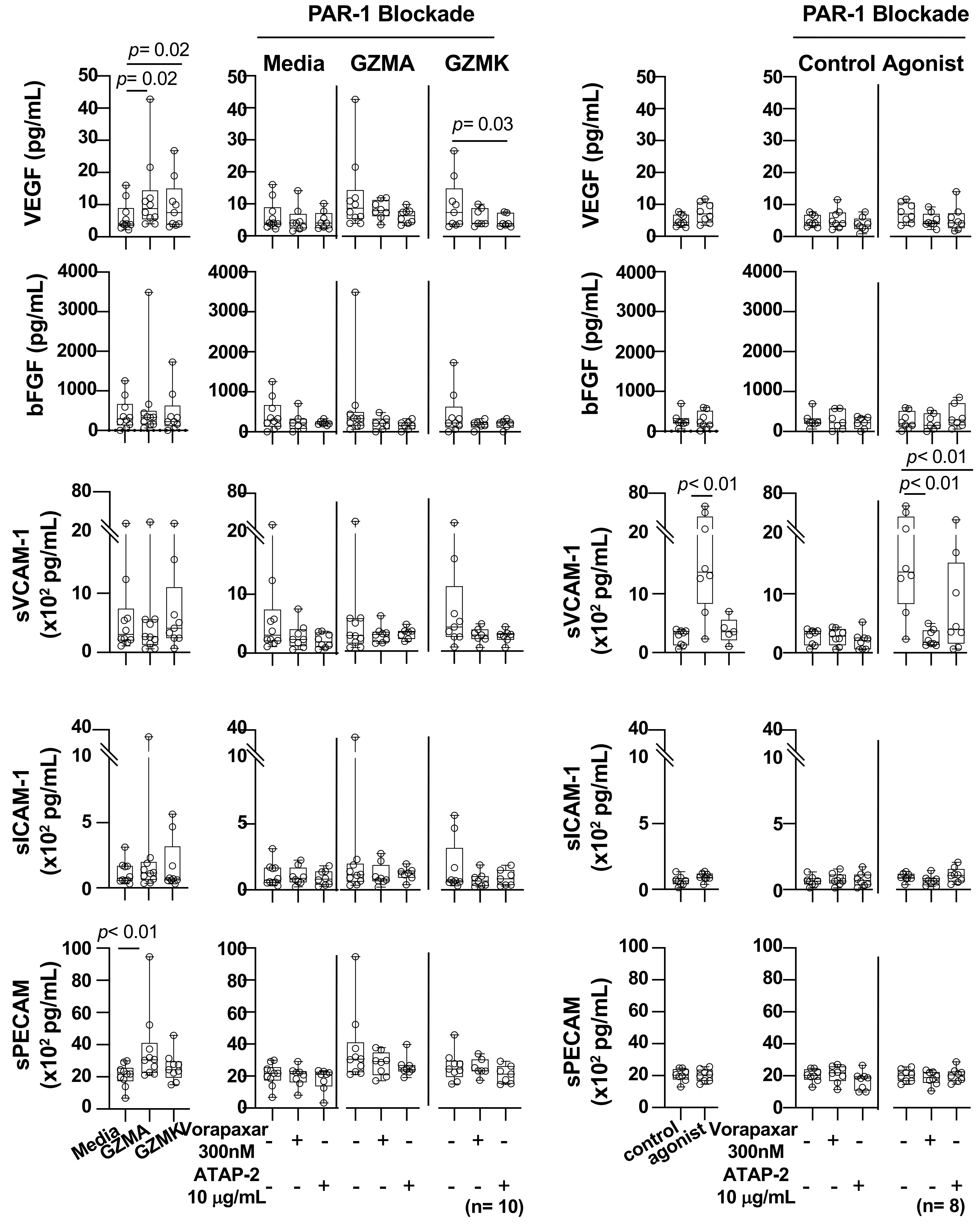
PAR-1 Blockade
PAR-1 Blockade
Media
GZMA
GZMK
Control Agonist
p= 0.02
p= 0.02
p= 0.03
p< 0.01
p< 0.01
p< 0.01
p< 0.01
VEGF (pg/mL)
bFGF (pg/mL)
sVCAM-1 (x10² pg/mL)
sICAM-1 (x10² pg/mL)
sPECAM (x10² pg/mL)
Media
GZMA
GZMK
control
agonist
Vorapaxar 300nM
ATAP-2 10 µg/mL
(n= 10)
(n= 8)
